# Elderly women with metabolic syndrome present higher cardiovascular risk and lower relative muscle strength

**DOI:** 10.1590/S1679-45082013000200007

**Published:** 2013

**Authors:** Darlan Lopes Farias, Ramires Alsamir Tibana, Tatiane Gomes Teixeira, Denis César Leite Vieira, Vitor Tarja, Dahan da Cunha Nascimento, Alessandro de Oliveira Silva, Silvana Schwerz Funghetto, Maritza Alves de Sousa Coura, Renato Valduga, Margô Gomes de Oliveira Karnikowski, Jonato Prestes

**Affiliations:** 1 Universidade Católica de Brasília, Brasília, DF, Brazil; 2 Universidade de Brasília, Brasília, DF, Brazil

**Keywords:** Metabolic syndrome X/complications, Risk factors/etiology, Cardiovascular diseases/prevention & control, Muscle strength, Aging

## Abstract

**Objective:**

To compare the metabolic, anthropometric, arterial blood pressure, and muscle strength parameters of elderly women with and without metabolic syndrome.

**Methods:**

A case-control study with 27 (67.3±4.8 years of age, 31.0±5.0kg/m^2^) elderly women with metabolic syndrome and 33 (68.8±5.6 years of age, 27.2±5.3kg/m^2^) sedentary control elderly women. They were submitted to an evaluation of body composition by means of dual-energy X-ray absorptiometry and muscle strength testing with 10 maximal repetitions of knee extension.

**Results:**

When compared to the elderly women without metabolic syndrome, those with the metabolic syndrome had higher levels for body mass (72.2±13.5 *versus* 63.4±14.6kg, p=0.03), body mass index (31.0±5.0 *versus* 27.2±5.3kg/m^2,^ p=0.007), fat mass (30.9±9.9 *versus* 24.4±8.5kg, p=0.01), systolic arterial pressure (125.1±8.2 *versus* 119.3±8.7mmHg, p=0.01), diastolic arterial pressure (75.5±6.9 *versus* 71.4±6.7mmHg, p=0.03), mean arterial pressure (92.5±6.2 *versus* 87.1±6.7mmHg, p=0.004), blood glucose (103.8±19.1 *versus* 91.1±5.9mg/dL, p=0.001), triglycerides (187.1±70.2 *versus* 116.3±36.7mg/dL, p=0.001), and creatine kinase (122.6±58.6 *versus* 89.8±32.5U/L, p=0.01); lower levels were found for fat-free mass (55.9±5.8 *versus* 59.3±6.7%; p=0.05), HDL-C (40.7±5.0 *versus* 50.5±10.1mg/dL, p=0.001), and relative muscle strength (0.53±0.14 *versus* 0.62±0.12, p=0.01).

**Conclusion:**

Elderly women with metabolic syndrome have a higher cardiovascular risk and less relative muscle strength when compared to those without metabolic syndrome. Relative muscle strength may be related to the cardiovascularr risk factors of the metabolic syndrome.

## RESUMO

**Objetivo:**

Comparar os parâmetros metabólicos, antropométricos, pressão arterial e força muscular de mulheres idosas com síndrome metabólica e sem síndrome metabólica.

**Métodos:**

Estudo caso-controle com 27 (67,3±4,8 anos 31,0±5,0kg/m^2^) idosas com síndrome metabólica e 33 (68,8±5,6 anos 27,2±5,3kg/m^2^) idosas controle sedentárias. As idosas foram submetidas à avaliação de composição por meio da absorciometria de raio X de dupla energia e teste de força muscular de 10 repetições máximas na extensão de joelhos.

**Resultados:**

Quando comparadas às idosas sem síndrome metabólica, as idosas com síndrome metabólica apresentaram índices maiores para: massa corporal (72,2±13,5 × 63,4±14,6kg; p=0,03), índice de massa corporal (31,0±5,0 × 27,2±5,3kg/m^2^; p=0,007), massa gorda (30,9±9,9 × 24,4±8, 5kg; p=0,01), pressão arterial sistólica (125,1±8,2 × 119,3±8,7mmHg; p=0,01), pressão arterial diastólica (75,5±6,9 × 71,4±6,7mmHg; p=0,03), pressão arterial média (92,5±6,2 × 87,1±6,7mmHg; p=0,004), glicemia (103,8±19,1 × 91,1±5,9mg/dL; p=0,001), triglicerídeos (187,1±70,2 × 116,3±36,7mg/dL; p=0,001) e creatina quinase (122,6±58,6 × 89,8±32,5 U/L; p=0,01); foram menores: massa livre de gordura (55,9±5,8 × 59,3±6,7 %; p=0,05); HDL-C (40,7±5,0 × 50,5±10,1mg/dL p=0,001) e força muscular relativa (0,53±0,14 × 0,62±0,12; p=0,01).

**Conclusão:**

Idosas com síndrome metabólica apresentam maior risco cardiovascular e menor força muscular relativa, quando comparadas às idosas sem síndrome metabólica. A força muscular relativa pode estar relacionada aos fatores de risco cardiovascular da síndrome metabólica.

## INTRODUCTION

Metabolic syndrome (MS) is characterized by cardiovascular risk factors, such as alterations in lipids, arterial hypertension, central adiposity, and insulin resistance. Epidemiological studies have shown strong associations between MS and the risk of developing cancer of the digestive system, diabetes, cardiovascular diseases, and early death^(1)^, besides a greater use of healthcare services and medical costs^(2)^. Data from the National Health and Nutrition Examination Survey^(3)^ informed that the rates of MS prevalence were 35.1% in men and 32.6% in women. In the central region of Brazil, Dutra et al.^(4)^ reported that the MS prevalence rates were 32%, for both males and females.

In this respect, changes in lifestyle and especially in the level of physical activity may help in the treatment and prevention of MS^(5)^. Additionally, the physical fitness components related to health, such as muscle strength and mass, play a significant role in carrying out motor tasks, decreasing the risk of falls, in addition to having repercussions in health, longevity, and quality of life of elderly people^(6,7)^. Consequently, some studies showed a possible association between muscle strength and decrease in cardiovascular risk factors^(8)^, obesity^(9)^, high blood pressure^(10)^, MS^(11)^, and early death^(7)^.

However, the studies that analyzed the association between muscle strength and MS only used young men and women^(11–14)^, and elderly British men^(15)^. It must be emphasized that aging is also associated with a progressive loss of muscle strength and mass^(16)^. We are not aware of any study that compares muscle strength of elderly Brazilian women with and without MS. The association of aging, which is a chronic degenerative process^(16)^, with MS may increase the risk for various comorbidities, which differentiates this population from young people with MS. Therefore, the initial hypothesis of the present study was that elderly women with MS would have less relative muscle strength and greater cardiovascular risk when compared to women without MS.

## OBJECTIVE

To compare the muscle strength of ten maximal repetitions (10 MR) on knee extension in elderly Brazilian women with and without MS, as well as to analyze if women at low risk showed differences in relative muscle strength.

## METHODS

A case-control study was performed during the period of August 2011 to July 2012, with volunteers recruited by public notice in local circulation newspapers and by telephone contact, based on a registry at the University, through the Center for Social Interaction of the Elderly (CCI, acronym in Portuguese). Volunteers were selected regardless of race or social class. The study was approved by the Ethics and Research Committee of the *Universidade Católica de Brasília* (UCB); the Informed Consent Form and questionnaire on readiness for physical activity (PAR-Q) were obtained from all volunteers before inclusion in the study. The sample was made up of 57 volunteers divided into two groups: 27 (67.3±4.8 years of age, 31.0±5.0kg/m^2^) elderly women with MS and 33 (68.8±5.6 years of age, 27.2±5.3kg/m^2^) without MS who did not engage in regular guided physical activity.

In order to be included in the study, the participants needed to have been at least 6 months with no regular physical exercise (PAR-Q), not be in hormone replacement therapy (self-reported), and be aged over 60 years. The exclusion criteria were (1) being incapable of walking without assistance; (2) having a unilateral or bilateral hip prosthesis; (3) being a smoker, and (4) having an abnormality in cardiac conduction or perfusion that would contraindicate the physical activity.

All the volunteers were submitted to the following tests before muscle strength evaluation: blood sample collection, dual-energy X-ray absorptiometry (DEXA), and blood pressure evaluation.

### Anthropometry

Measurements were made to enable establishing the anthropometric profile defined by the body mass index (BMI). For body composition assessment, the percentages of lean mass and fat mass were analyzed by means of DEXA (DEXA, General Electric-GE model 8548 BX1L, 2005, Lunar DPX type, Encore 2005 software). The tests included a complete body scan of the volunteers, in supine position, during approximately 17 minutes, with the apparatus always regulated and operated by a technically trained professional.

### Biochemical analysis

The individuals were submitted to absolute rest for 30 minutes; next, a 20mL sample of blood was collected from the antecubital vein by means of venous puncture, using the vacutainer system with EDTA anticoagulant, allowing determination of triglycerides by the enzymatic-colorimetric method (GPO/POD) on Autohumalyzer equipment (Human GMBH, Germany). HDL-C was determined by ionic exchange followed by colorimetric reaction with the Linco^®^ Research Inc. kit (St Louis, USA), and blood glucose by hexokinase enzymatic assay. C-reactive protein (CRP) and creatine kinase (CK) were determined by turbidimetry methodology, intensified with particle reaction by the Cobas Mira Plus spectrophotometer (Roche Diagnostic, GmBH -Germany), with Biosystem (Bayer^®^) calibrator and serum control. Cortisol was dosed by electrochemiluminescence following specifications of the kit. The technique was developed on the Elecsys 2010 Roche Diagnostics device.

### Resting arterial pressure

Determination of systolic arterial pressure (SAP) and diastolic arterial pressure (DAP) was performed by the oscillometric system, adopting the methodology proposed by the 5^th^ Brazilian Guideline for Arterial Hypertension^(17)^ with an oscillometric measurer (Microlife 3AC1-1, Widnau, Switzerland), validated by the European Society of Hypertension. With the elderly woman in sitting position, after 10 minutes of rest, the right arm supported at heart level, with cuff appropriate for the arm size, the cuff of the device was placed about 3cm above the antecubital fossa centralizing the rubber pouch over the humeral artery. The SAP and DAP values measured were used to calculate the mean arterial pressure (MAP) using the equation MAP = DAP+ [(SAP - DAP) ÷ 3].

### Determining MS

The MS was identified considering the parameters defined by the First Brazilian Guideline for Diagnosis and Treatment of Metabolic Syndrome^(18)^, which is based on the criteria defined by the National Cholesterol Education Program's - Adult Treatment Panel III (NCEP - ATP III). According to NCEP - ATP III, MS represents the combination of at least three of the five parameters used to define MS: high abdominal circumference (in the present study we used the criterion of BMI ≥30kg/m^2^), increased triglycerides (≥150mg/dL), low HDL-cholesterol (<50mg/dL), high fasting blood glucose (≥110mg/dL) or diabetes, and increased blood pressure (systolic pressure ≥130mmHg and/or diastolic pressure ≥85mmHg, or use of anti-hypertensive agents).

### Muscle strength test

The elderly women went through a session to become familiar with knee extension, with three series of 10 submaximal repetitions. Seventy-two hours later, the 10 MR test was performed on the extension chair (Cybex International, Medway, MA) as per the following recommendations: (1) warm-up of 5 to 10 repetitions with intensities of 40 to 60% of an estimated maximal repetition (1 MR); (2) rest for one minute, followed by 3 to 5 repetitions with 60% of the estimated 1 MR, and rest for 3 minutes; (3) weight increment trying to reach 10 MR in 3 to 5 attempts, using 5 minutes of interval between one attempt and another, and 10 minutes between exercises; (4) the value recorded was for 10 repetitions, with the maximal weight lifted on the last successful attempt. To determine the reliability of the 10 MR tests, two tests were applied, with a 48-hour interval^(19)^. Relative muscle strength was calculated by means of the following formula: relative strength = absolute strength (kg)/body mass (kg)

### Statistical analysis

The significance level for all variables studied was p≤0.05. Initially, the descriptive analysis of the sample was made with measurements of central tendency and dispersion. Next, the Kolmogorov-Smirnov test was performed to evaluate normality of the data and, according to the results, Student's non-paired t test was used for parametric data, or the Mann-Whitney test for non-parametric data. Additionally, one-way ANOVA was used with the Bonferroni correction to compare the relative muscle strength among the elderly women with or without a risk factor for MS, two risk factors for MS, and more than three. Also performed was the correlation between relative muscle strength and cardiovascular risk factors, anthropometric factors, and biochemical factors, using Pearson's correlation. Data were analyzed using Statistical Package for the Social Sciences (SPSS) software, version 17.0. Assuming an effect size of 0.97 between the groups (based on a pilot study), a minimal sample of 11 volunteers for each group was required to allow an 85% power for the study for relative muscle strength.

## RESULTS

**Figure 1:**
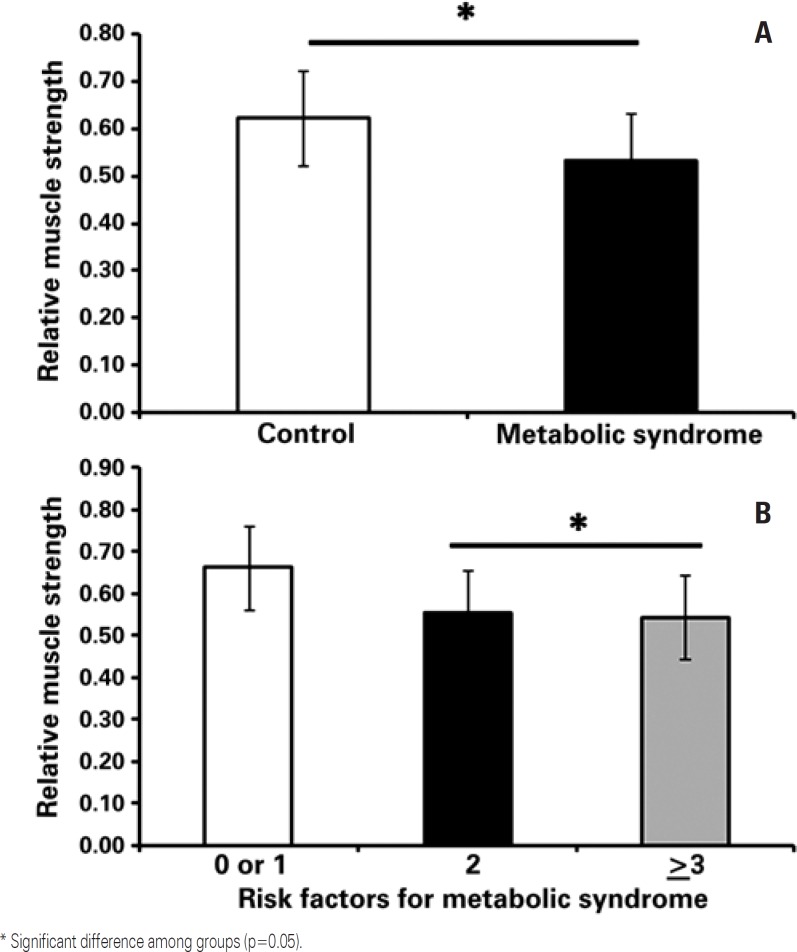
Relative muscle strength for the groups. (A) With and without metabolic syndrome; (B) none or one (n=18) risk factor for metabolic syndrome; two (n=18) risk factors for metabolic syndrome; and three (n=21) risk factors for metabolic syndrome

Anthropometric, blood pressure, biochemical, and inflammatory parameters of the elderly women with and without MS are shown on table 1. No significant differences were observed for age (p=0.30), height (p=0.96), and cortisol (p=0.57). Nevertheless, when compared to the women without MS, those with MS had higher values for body mass (p=0.03), BMI (p=0.007), percentage of fat mass (p=0.05), fat mass (p=0.01), fat-free mass (p=0.01), SAP (p=0.01), DAP (p=0.03), MAP (p=0.004), glucose (p=0.001), triglycerides (p=0.001), CK (p=0.01), and CRP (p=0.01). The percentages for fat-free mass (p=0.05), HDL-C (p=0.001), and relative muscle strength (p=0.01) were lower. Additionally, the elderly women who did not have risk factors or had one risk factor for MS were significantly stronger than those who showed two or more risk factors for MS (Figure 1).

**Table 1:** Anthropometric, blood pressure and biochemical parameters of women with and without metabolic syndrome

	MS (n=24)	Without MS (n=33)	p value
Age (years)	67.3±4.8	68.8±5.6	0.30
Height (m)	1.52±0.06	1.53±0.06	0.96
Body mass (kg)	72.2±13.5	63.4±14.6*	0.03
BMI (kg/m^2^)	31.0±5.0	27.2±5.3*	0.007
Fat mass (%)	44.1±5.8	40.7±6.7*	0.05
Fat mass (kg)	30.9±9.9	24.4±8.5*	0.01
Fat-free mass (%)	55.9±5.8	59.3±6.7*	0.05
Fat-free mass (kg)	38.0±4.2	35.1±4.1*	0.01
SBP (mmHg)	125.1±8.2*	119.3±8.7*	0.01
DBP (mmHg)	75.5±6.9*	71.4±6.7*	0.03
MBP (mmHg)	92.5±6.2	87.1±6.7*	0.004
TGL (mg/dL)	187.1±70.2	116.3±36.7*	0.001
Glucose (mg/dL)	103.8±19.1	91.1±5.9*	0.001
HDL (mg/dL)	40.7±5.0	50.5±10.1*	0.001
CRP (pg/mL)**	0.30	0.19*	0.01
CK (U/L)	122.6±58.6	89.8±32.5*	0.01
Cortisol (*μ*g/dL)	145±4.6	15.3±5.8	0.57

* Significant difference between the groups;

** Data expressed as median.

BMI: body mass index; SBP: systolic blood pressure; DBP: diastolic blood pressure; MBP: mean blood pressure; TGL: triglycerides; HDL: high-density lipoprotein; CRP: C-reactive protein; CK: creatine kinase; MS: metabolic syndrome.

The correlations between relative muscle strength and the other variables in both groups are shown on table 2. Inverse correlations were seen for relative muscle strength with fat mass and fat-free mass in the group without MS. On the other hand, in the group with MS, relative muscle strength was inversely correlated with body mass and BMI.

**Table 2:** Correlation among variables and relative muscle strength in women with and without metabolic syndrome

	Without MS		With MS	
	R	p value	R	p value
Age (years)	-0.07	0.10	-0.14	0.58
Body mass (Kg)	-0.33	0.11	-0.47	0.05*
Height (m)	-0.27	0.19	0.08	0.71
BMI (Kg/m^2^)	-0.30	0.15	-0.57	0.01*
Fat mass (%)	-0.47	0.01*	-0.46	0.06
Fat-free mass (%)	0.47	0.01*	0.46	0.06
Fat mass (kg)	-0.39	0.05*	-0.45	0.06
Fat-free mass (kg)	-0.19	0.35	-0.28	0.26
SBP (mmHg)	0.19	0.37	0.03	0.88
DBP (mmHg)	0.15	0.45	0.27	0.29
MBP (mmHg)	0.18	0.39	0.21	0.40
TGL (mg/dL)	-0.21	0.31	0.04	0.88
HDL (mg/dL)	0.15	0.46	0.00	0.98
Glucose (mg/dL)	0.11	0.60	-0.11	0.66
Cortisol (*μ*g/dL)	-0.05	0.80	-0.14	0.58
CK (U/L)	-0.15	0.47	0.24	0.34
CRP (mg/dL)	-0.09	0.64	0.30	0.23

* Significant correlation (p<0.05).

BMI: body mass index; SBP: systolic blood pressure; DBP: diastolic blood pressure; MBP: mean blood pressure; TGL: triglycerides; HDL: high-density lipoprotein; CK: creatine kinase; CRP: C-reactive protein; MS: metabolic syndrome.

## DISCUSSION

Confirming the original hypothesis, the women with MS showed a higher cardiovascular risk and less relative muscle strength when compared to those without MS. Additionally, the results demonstrated that women with two or more risk factors for MS had less relative muscle strength when compared to those who had no risk factor or had one risk factor for MS.

The Framingham Offspring study^(20)^, which included 2,406 men and 2,569 women aged between 19 and 74 years, examined the grouping of metabolic and cardiovascular factors relative to the risk of coronary artery diseases (CAD). The risk factors considered were HDL-C, BMI, SAP, triglycerides, glucose, and total cholesterol. The authors showed that the participants with three or more risk factors increased their risk of developing CAD 2.4 times for men and 5.9 times for women when compared to volunteers with no risk factor. Forti et al.^(21)^ demonstrated that the probability all causes of mortality in elderly women with MS (70 to 79 years) was approximately twice as high as that in women without MS.

Therefore, modifications in lifestyle, especially in terms of engagement in physical activities, can help as a non-drug instrument in treating risk factors for MS in elderly women. Despite the fact that cardiorespiratory aptitude is associated with all causes of mortality and cardiovascular diseases^(5)^, recent studies have demonstrated that muscle strength and mass are aspects that play an important role in performing daily tasks, longevity, and quality of life, especially in elderly individuals^(6,7)^. Also demonstrated was an association between muscle strength and a decrease in cardiovascular risk factors^(8)^, obesity^(9)^, high blood pressure^(10)^, metabolic syndrome^(11)^, and early death^(7)^.

The results of the present study are in agreement with those presented by Tibana et al.^(11)^, which demonstrated that middle-aged Brazilian women with MS showed less relative muscle strength when compared to women without MS. Similar results were presented by Miyatake et al.^(13)^ after analyzing Japanese middle-aged men with and without MS. The study revealed that individuals with MS had less relative manual grip strength and lower limb strength when compared to the control group. Jurca et al.^(12)^ and Wijndaele et al.^(14)^ found a significant association between low values of muscle strength and the development of MS in women.

However, as far as we know, the study by Sayer et al.^(15)^ was the only one developed for elderly persons with or without MS. Similar to the results shown in the present study, the elderly women with MS showed less relative muscle strength when compared to those without MS. These results increase the concern about elderly women with MS, since the very process of aging causes a significant reduction of muscle strength and mass, a process that compromises the performance of daily activities^(16)^.

Few studies have analyzed the possible mechanisms associated between loss of muscle strength and mass and metabolic dysfunctions. It is known that skeletal muscle is an important determinant of the resting metabolic rate^(22)^ and a target-organ for the storage of glucose^(23)^. Also, Izumiya et al.^(24)^ used genomic intervention to produce muscle hypertrophy in the muscle fibers of obese mice. The animals that had their muscle fibers hypertrophied by the transgenic induction of Akt1 (mediator of muscle hypertrophy) exhibited reductions in body weight, in fat mass, blood glucose, insulin, and leptin. When there was transgenic inactivation of the Akt1 route, the animals lost muscle mass, which was associated with an increase in body fat deposition and circulating concentration of leptin and insulin, indicating a metabolic dysfunction. In this aspect, it is plausible to assume that the elderly women with MS in the present study had a lower percentage of fat-free mass when compared to those without MS, which may be associated with the metabolic dysfunctions and less relative muscle strength. However, this study has a cross-sectional case-control characteristic, which impedes the establishment of a cause and effect relation. Thus, longitudinal and intervention studies are necessary in order to discover the possible mechanisms of muscle strength and mass related to MS. Other limitations of this study include the reduced number of participants and the use of medications that affect metabolism which are commonly used to treat MS.

Furthermore, the elderly women with MS showed higher concentrations of CK when compared to those without MS. The levels of CK were associated with the development of hypertension^(25)^, and as far as we know, no study has analyzed the levels of CK in elderly women with MS. In this respect, Brewster et al.^(26)^ demonstrated that in spontaneously hypertensive rats, the values of CK in the left ventricle and aorta are greater than in normotensive rats. Therefore, the increase in CK may be associated with vasoconstriction, and with increased vascular reactivity and cardiac contractility. However, other factors may have affected the circulating concentrations of CK, such as the use of statins to control hypercholesterolemia, since this medication may cause rhabdomyolysis, with acute renal failure and even death^(27)^.

As per this aspect, the inclusion of the resistance training in elderly women seems to be clinically relevant, since besides increasing strength and functional capacity^(28)^, it induces alterations beneficial for the cardiovascular and metabolic system^(29)^ in elderly women. Additionally, data from this study group demonstrated that the resistance training does not induce a systemic increase in inflammatory cytokines in women with MS after an acute resistance exercise session^(30)^, besides chronically decreasing arterial pressure and resistin (data not published). Nonetheless, a single resistance exercise session is capable of reducing arterial pressure during 24 hours in overweight and obese women^(31)^.

## CONCLUSION

In conclusion, elderly women with MS demonstrated less relative muscle strength and greater cardiovascular risk. Furthermore, elderly women with two or more risk factors for MS had less relative muscle strength when compared to those with no risk factor or with one risk factor for MS, which increases concern with health care. Additionally, the present results demonstrated that the elderly women with MS had a higher concentration of CK.
